# Optimization of the Extraction Methodology of Grape Pomace Polyphenols for Food Applications

**DOI:** 10.3390/molecules28093885

**Published:** 2023-05-04

**Authors:** Joana Moutinho, Irene Gouvinhas, Raúl Domínguez-Perles, Ana Barros

**Affiliations:** 1 Centre for the Research and Technology of Agro-Environmental and Biological Sciences (CITAB)/Institute for Innovation, Capacity Building and Sustainability of Agri-Food Production (Inov4Agro), University of Trás-os-Montes and Alto Douro (UTAD), 5000-801 Vila Real, Portugal; joanamoutinho23@gmail.com; 2 Phytochemistry and Healthy Food Lab (LabFAS), Department of Food Science and Technology, CEBAS, CSIC, University Campus of Espinardo-25, 30100 Murcia, Spain; rdperles@cebas.csic.es

**Keywords:** winery by-products, phenolic extraction, colorants, response surface methodology, antioxidant capacity

## Abstract

This study aims to take advantage of the wine industry by-products and extract bioactive compounds from grape pomace by applying methodologies susceptible to be integrated easily into industrial workflows because of the association with standard instrumentation and facilities, while the main factors affecting the efficiency of the process have been optimized. The sampling consisted of two grape varieties: ‘Touriga Nacional’ and ‘Sousão’. A response surface methodology (RSM) method was used to optimize the extraction conditions based on three independent variables according to the chemical characteristics and stability/lability traits associated with polyphenols; the main bioactive phytochemical in grape pomace: solvent (50%, 70%, and 90% ethanol); temperature (20 °C, 40 °C, and 60 °C); and pH (0.5% HCl, 2% HCl, and 3.5% HCl). The phytochemical profile, as well as the radical scavenging and reducing powers were determined on 27 different samples. The highest yield and antioxidant activity corresponded to extracts obtained at 60 °C using 3.5% HCl and 70% ethanol. The values for total phenols and flavonoids were 44.93 mg of gallic acid equivalents (GAE) and 22.95 mg of catechins equivalents (CE) per gram, respectively. Concerning the evaluation of antioxidant capacity using various assays such as ABTS, DPPH, and FRAP, the results obtained were 0.30, 0.43, and 0.36 mmol of Trolox equivalent antioxidant capacity (TEAC) per gram, correspondingly. The analysis of the extract obtained with the best extraction performance using these parameters via High-Performance Liquid Chromatography–Mass Spectrometry has been also performed, allowing us to identify fourteen (14) compounds, including phenolic acids (*n* = 3), flavonols (*n* = 7), and anthocyanins (*n* = 4). As a result of this process, the best conditions for the production of a natural and environmentally friendly dye, not only avoiding waste but also reusing these by-products, were achieved.

## 1. Introduction

The relevance of the agro-food sector producing high quantities of waste is well represented by the wine industry, which is responsible for the production of thousands of tons of by-products (solid, liquid, and semisolid). In this regard, about 75% of the 27 million tons of grapes (*Vitis vinifera*) produced in Europe are used in winemaking [1]. As a result of the winemaking activity, on average, around 20% of the grape material gives rise to a by-products subsidiary of wine production, the known grape pomace [2,3]. Hence, grape pomace consists mostly of skins, seeds, and some stalks, and is considered a biodegradable solid mixture obtained after the grape pressing to extract the must [4]. Fine-tuning the processing settings needed for achieving new valorization alternatives that turn grape pomace into new added-value co-products in the light of the “circular economy” concept would allow us to obtain new and safe sources of bioactive polyphenols (phenolic acids, stilbenes, catechin-derivatives and proanthocyanidins, flavonols, and anthocyanins) that include a range of individual compounds with potentially synergic functional capacities [5]. Indeed, these compounds have been demonstrated as responsible for valuable biological properties that contribute to human health [1,6,7]. To the present date, because of these biological features, among the diverse valorization alternatives, grape pomace has been highlighted in terms of its antioxidant, anti-inflammatory, and antimicrobial activities, which confers to it preventive potential against an array of pathophysiological conditions [8,9]. In this regard, recent studies have already demonstrated, in vitro, important biological traits of grape pomace’s phytochemicals, namely radical scavenging and reducing power, anti-inflammatory activity in cell lines, such as in alveolar epithelial type I cells (R3/1 cell line), resorting to the interaction with the translocation factor NF-κB, as well as antibacterial activity against several species and strains of medical interest, e.g., *Staphylococcus aureus* and *Enterococcus faecalis* [10,11,12]. Based on these biological capacities, functional extracts from these materials would help to replace, to some extent, or reduce the concentration of synthetic products nowadays used as preservatives in manufactured food products, whose effects on human health is under discussion [13]. Thus, to take advantage of the polyphenolic fraction of this matrix and advance towards an efficient valorization strategy, optimizing extraction conditions remains crucial; specially applying methodologies which scale up to the industrial level is feasible, since this approach will allow for the recovery of higher percentages of polyphenols relative to the currently applied procedures and conditions. In this regard, the solvent selection is the main factor affecting extraction efficiency [14]. Indeed, the use of hydroethanolic solvents is the most suitable alternative given the chemical properties of polyphenols that include compounds with different polarity [6,15], also ensuring the application of the extracts in the food industry, since this solvent is recognized as safe by the European Food Safety Authority (EFSA) [16].

Furthermore, due to the environmental problems emerging over the last decades, saving the environment is increasingly urgent. Based on the circular economy paradigm, the reuse of by-products from the agro-food industry in general (and specifically referred to the wine industry) as a sustainable source of bioactive compounds constitutes a valuable alternative to enhance the obtaining of added-value co-products from these residues that, otherwise, would be disposable and heavily harmful for the local environments. With this objective, the use of a Response Surface Methodology (RSM) arises as an approach that allows for minimizing the number of experiments carried out to get the optimal conditions and, on the other hand, lower costs and resources are needed [15,17]. In the selection of the most appropriate parameters to be evaluated, the present work has chosen a combination of factors that complement previous optimizations described in the literature in the recent years by Da Porto et al. and Casagrande et al. [17,18] that were focused on fine-tuning the setting for solvent concentration, extraction time, type of solvent, and the influence of the liquid/solvent ratio. Hence, the complementarity of this work with the previous descriptions in the literature is based in the close effect of pH on the stability of polyphenols [19], the determination of which will complete the data needed to set up the optimal conditions for valorizing grape pomace as a source of bioactive polyphenols.

Based on these premises, the present study aimed to optimize liquid–solid extraction from grape pomace (including skin, seeds, and stems) of two Portuguese red varieties, ‘Touriga Nacional’ and ‘Sousão’, by evaluating the influence of three independent variables, the solvent (ethanol) percentage (% Et, *X*1; 50%, 70%, and 90% ethanol); pH (percentage of hydrochloric acid) (%HCl, *X*2; 0.5% HCl, 2% HCl, and 3.5% HCl); and extraction temperature (°C, *X*3; 20 °C, 40 °C, and 60 °C), by using RSM. An additional factor closely related to the efficiency of the polyphenolic extraction is the solid/solvent ratio; nonetheless, this has been broadly described in the literature demonstrating that, for maceration, the augment of the solid-to-solvent ratio increases the polyphenolic yield significantly up to 1:20 and non-significantly up to 1:60 [20]. Thereby, to ensure the highest yield while saving solvent according to the industrial procedures and previous results retrieved in our lab for grape pomace, in the present study a ~1:40 ratio was established. The efficiency of the extraction conditions assayed was set up on the base of the concentration of phenolic acids and flavonoids, as well as the radical scavenging and reducing power (ABTS, DPPH, and FRAP). The extract obtained using the best extraction conditions was also assessed on the individual phenolic content via High-Performance Liquid Chromatography coupled to a diode array and electrospray ionization mass spectrometry detectors (HPLC–DAD–ESI-MS/MS).

## 2. Results and Discussion

### 2.1. Yield of the Assayed Extraction Conditions

Table 1 shows theoretical and experimental values regarding the evaluation of various parameters, such as TPC, FC, ABTS, DPPH, and FRAP. When comparing the theoretical values with the experimental results, it was observed that both of them were close to one another for each extraction condition under evaluation.

The highest TPC and FC was verified when carrying out the extractions at the pH achieved in solvents supplemented with HCl at 3.5% and 60 °C, using 70% of ethanol concentration (Table 1). Beyond this, in good agreement with the preponderant antioxidant responsibility of these compounds, these extraction settings also provided the best results for the radical scavenging power (0.30 mmoles TEAC/g dw for both ABTS and DPPH methods, on average) and the reducing capacity (0.36 mmoles TEAC/g dw for the FRAP method). In the selection of parameters to be optimized for enhanced extraction of phenolic compounds of grape pomace, the enhanced extracting capacity of ethanol is in good agreement with previous descriptions that inform on the close relationship between the solvent type and the extraction efficiency [14]. Indeed, the choice of ethanol became sound scientifically as well as practically, since it is recognized as a safe solvent for the food industry [16] as well as an eco-friendly organic solvent capable of extracting high percentages of polyphenols [21]. The preference for using water in the mixture with the organic solvent is also one of the ways that is recognized to get greater extraction efficiency. In this regard, the presence of water increases the permeability of cell tissue [14], although it could become ineffective at low temperatures [8].

According to these results, the extraction conditions providing the lowest efficiency in terms of TPC, FC, and antioxidant capacity was pH 0.5, at 20 °C, and 90% of ethanol; and the pH when adding 2.0% HCl, at 20 °C, using 70% of ethanol concentration for flavonoids (Table 1). According to the literature, the lowest concentrations and radical scavenging capacities are obtained when applying the lowest temperature and percentages of HCl (pH of the extraction solvent), while, on the contrary, the power to extract phenolics and thereby obtain higher radical scavenging capacities increases with temperature [21]. In this regard, Morelli and Prado (2012) [22] described that the best results were obtained with temperatures between 40 °C and 60 °C. The use of higher temperatures allowed enhanced solubilities, thereby improving the efficiency of the extraction process [23].

The pH of the solution is another important parameter in this study; the choice of acidifying the solvent with HCl is an alternative used to achieve a better selection of compounds to be extracted [14]. In this regard, to extract anthocyanin pigments from black carrots, Türker and Erdoğdu (2006) analyzed the impact of pH on the stability of the pigments, reporting that the coloured flavonoids are more stable in more acidic environments (pH = 2) [24]. Thus, with increasing pH, the stability of the pigments is compromised. As well, during the storage period, a lower pH offers stability to the samples. Correspondingly, the antioxidant and reducing powers of the extracts obtained are also lower in comparison with samples obtained using extraction solvents that featured more acidic pH (between 3 and 5) [25].

In addition, Librán et al. (2013) examined the extraction of the phenolic fraction of wine residues, reporting the relative influence of the pH and solvent on the final concentration of phenolics [26]. The detailed analysis of various parameters allowed them to identify acid pH and high percentages of ethanol as critical factors to retrieve the best values.

### 2.2. Model Fitting

**Figure 1:**
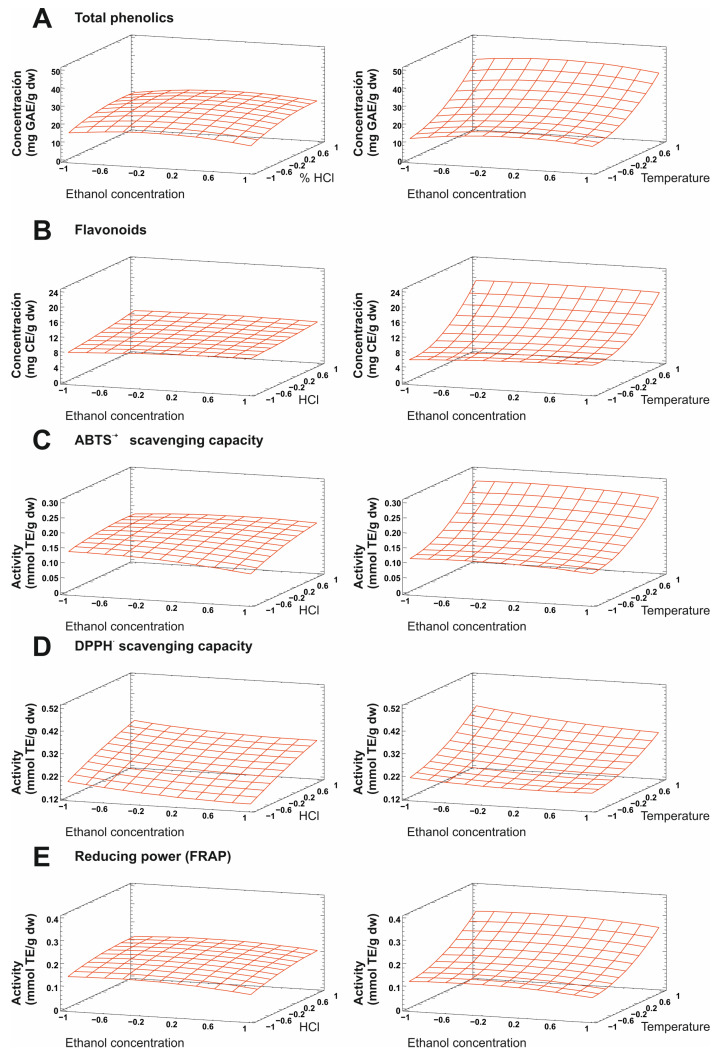
Response surface plots showing the effect of ethanol concentration, HCl percentage, and extraction temperature on the extraction of grape pomace’s polyphenols and the antioxidant capacity. Total phenolic compounds (**A**), flavonoids (**B**), ABTS^●+^ scavenging power (**C**), DPPH^●^ scavenging power (**D**), and reducing capacity (**E**).

The regression coefficients of the intercept, linear, quadratic, and interaction terms of the model were calculated using the least squares method. The effect of the linear, quadratic, or interaction coefficients on the response for the separate variables (TPC and FC, as well as the DPPH^•^ and ABTS^•+^ scavenging capacity and the reducing power) was studied using analysis of variance (ANOVA) (Figure 1 and Table 2).

The models obtained from the RSM methodology for total phenolics and flavonoid content presented coefficients of regression (R^2^) ranging from 0.869 to 0.962, demonstrating that at least 87% of the variation in the dependent variable can be explained by the variation in the independent variables (Table 2). The R^2^ for total phenolics was 0.962, meaning that the model almost adequately fits the total phenolics content. As for flavonoids, a value of 0.909 was obtained. In regard to the antioxidant capacity, RSM-based models enabled to ensure a high ABTS and DPPH antiradical activity, as well as FRAP-based reducing capacity, displaying R^2^ coefficients of 0.945 for FRAP, 0.958 for DPPH, and 0.975 for ABTS. These results support the reliability of the optimization procedure.

Statistical parameters, such as the R^2^, F-value, or Mean Absolute Error (MAE), were used to determine the fitting between the theoretical and experimental data and the experimental design. The regression coefficient (R^2^) evidenced the goodness of fit, being the coefficient obtained in the present work (>0.900) indicative of high goodness of fit and power of prediction. *p*-values < 0.05 inform on the significant influence of the evaluated factors over the dependent variables monitored [27]. Given the values of the model’s indicators presented for the variables considered in the matrix under study (grape pomace), it can be concluded that they are consistent results, thus informing that the model is well-adjusted.

The highest experimental values for phenolic content and antioxidant capacity were observed when using the highest temperature (60 °C). These results seem to be a consequence of the increased solubility when using high temperatures, which in turn makes the mass transfer faster. The penetration of the solvent into the matrix at a high temperature occurs more easily due to the reduction of its viscosity [14]. Additionally, Rajha et al. (2014) quantified the best extraction conditions for anthocyanins and tannins in wine by-products using the RSM [23]. Throughout the various analyses, the tendency observed appeared as a robust response to the extracting conditions assayed. This is not only a benefit for the implementation of these parameters at the industrial scale but is also a way to save costs when the best conditions are not only high temperatures but also the reduction of the extraction time, allowing for the prevention of the degradation of the phenolic compounds. On the opposite, the longer extraction time in combination with high temperatures provided lower efficiencies, tentatively due to the oxidation suffered by the phenolic compounds under these time–temperature combinations [23].

In another study, Bucić-Kojić et al. (2009) used the grape seeds as a matrix, allowing for the evaluation of the antioxidant capacity and color of the extract based on different temperatures and ethanol percentages during extraction [28]. In this case, the temperature ranged from 25 to 80 °C and the ethanol percentages varied from 50% to 96%. A greater extractability was obtained when applying the highest temperature; however, when developing extractions at 25 °C, the efficiency was significantly lower. Beyond the concentration of bioactive polyphenols, the evaluation of the antioxidant capacity provided results that were interpreted in the same way. Thus, the use of higher temperatures during the extraction provides better values. Regarding the use of ethanol in the extraction, according to the values for TPC, the best results were obtained with 50% ethanol (129.59 mg/g dw), while the extractions performed using only water provide lower values (30.87 mg/g dw). According to these results, it can be stated that the use of a solvent that has both water and ethanol in its composition is much more effective than a solvent with either only water or only ethanol. Similarly, Bucić-Kojić et al. (2009) in the HPLC analysis achieved the best results regarding the efficiency of the extraction with 50% ethanol [28]. These results are in good agreement with the information available in the literature that describes the higher leaching of polyphenols in plant materials into hydroalcoholic solvents because of the presence of compounds with different polarities depending on the chemical traits; e.g., glycosilation [29]; this higher leaching goes beyond the plant material–solvent interaction that strongly conditions the leaching of the target compounds into the extraction solvent [30].

When evaluating the results regarding the pH of the extraction solvent in the present work, the best values were achieved with the highest percentage of HCl (3.5%), providing the lowest pH. In this regard, it has been broadly described that the extraction of phenolic compounds, such as flavonols and total phenols, is more efficient under more acid pH conditions [9]. Except for flavonoids, when using 50% food-grade ethanol, the best results were retrieved when supplementing extracting solvents with 2% HCl instead of the higher percentage. Thereby, to obtain a higher yield, the use of ethanol in lower concentrations jointly with a higher pH value provided the best results, and was thus identified as the most appropriate condition [26].

In recent years, Barros et al. (2015) optimized the extracting conditions for the bioactive polyphenols of grape stems, another promising residue from the winery industry with the potential to be valorized as a source of antioxidants, of special interest due to the large scale that it is capable of generating and its undervaluation compared to other by-products generated by this industry [9]. According to the optimum pH, relative to the target compounds to be extracted, TPC or FC require a lower supplementation of extraction solvents with HCl (between 2.0% and 3.9%). Additionally, temperature is another important factor that needs to be optimized for enhanced extraction of polyphenols, since the different values for this variable may be associated with advantageous effects on the physical features of the matrix or harmful effects, and, as a result, harmful on the efficiency of the polyphenols’ extraction. Beyond this, although several compounds become unstable at high temperatures, Barros et al. used high temperatures in the extractions that, in combination with a short extraction length, would maintain the structural stability of the compounds extracted, which was hypothesized to be due to the physical properties of the plant material and the close relationship of this trait to the release of the phenolic compounds and the actual exposition to the high temperature featuring the extraction conditions [9].

Concerning the percentage of food ethanol in the optimization of the extraction, in the content of TPC, ABTS, and FRAP, the optimal percentage found in the present work was 70%. However, concerning the DPPH-based antioxidant capacity, the range of 50% to 70% provided the best results. Finally, concerning FC, the highest yield was obtained using 70% food-grade ethanol. In the optimization of the best conditions in the extraction of polyphenols in grape stems and seeds, Karvela et al. (2011) were supported by three factors at the time of extraction (time, pH, and percentage of ethanol) [31]. Three different grape varieties were tested, and several parameters were evaluated at the phenolic level. Based on the results obtained, Karvela et al. (2011) concluded that, for the extraction of flavonols, using 60% ethanol is the most suitable extracting condition [31]. On the other hand, when optimizing the extraction of flavones, the optimum ethanol percentage was 40%. The use of acidified solvents in conjunction with high temperatures during extraction is one way to separate the phenolic compounds that are extractable from the matrix under study. In this regard, for traditional extractions, the phenolic yield obtained was highest at a temperature between 60–80 °C. On the contrary, when applying alternative extraction technologies, such as subcritical water extraction (SWE), the optimal temperature is as high as 100–200 °C [32,33,34]. However, many studies have demonstrated that polyphenols are thermolabile compounds whose concentration decreases when applying temperatures above 80 °C [32,33,34]. Beyond temperature, other factors also are involved in the stability of polyphenols, namely pH. This factor strongly conditions the activity of key enzimes related to the stability of polyphenols, such as glycosidases, polyphenol oxidases, and/or peroxidases found in plant tissue [35]. In this regard, the stability of these enzymes depends on the pH [36], and, thereby, the joint consideration of both temperature and pH during the extraction process is critical for obtaining maximized polyphenolic yields. Moreover, the pH of the extraction media is very important for modulating the temperature effect. In this aspect, upon investigating the relationship between temperature and pH in the course of polyphenolic extractions, Havlíková and Míková described that, at temperatures ranging between 50–60 °C (the temperature set up as optimal in the present work for extracting grape pomace’s polyphenols), a low pH is critical in anthocyanin’s stability. Alternatively, at temperatures higher than 70 °C, no significant influence between pH and polyphenolic yield is observed [37].

Casagrande et al. (2019) also investigated the effect of extraction conditions (temperature (40 and 60 °C), time (15 and 45 min), and type of solvent (ethanol and acetone)) of TPC as well as the antioxidant activity of the grape pomace [18]. In this work, the TPC and radical scavenging activity of the grape pomace extract varied from 17.91 to 35.10 mg GAE/g dw and from 65.12 to 149.27 μmol TEAC/g dw, respectively. From this study, the authors concluded that acetone at 60 °C during 15 min was the best choice. Another study also investigated the optimization of the extraction of TPC, FC, anthocyanins, and proanthocyanidins and catechin derivatives from grape pomace via the RSM, in which the independent variables selected were the ethanol concentration (40, 65, and 90%), extraction time (6, 15, and 24 h), and liquid-to-solvent ratio (10:1, 30:1, and 50:1, L/S). The optimal extraction conditions were: 57% EtOH, 17 h, 50:1 L/S for TPC; 57% EtOH, 13 h, 50:1 L/S for FC; 62% EtOH, 16 h, 50:1 L/S for proanthocyanidins and catechins; and 52% EtOH, 6 h, 10:1 L/S for anthocyanins [17]. The present study and the previous reports described are in good agreement with the utility of the Box–Behnken experimental design as a valuable tool to estimate the effect of several extraction conditions for optimizing the yield of the bioactive polyphenols of different by-products.

### 2.3. Validation of the Predictive Models Developed

The equations obtained from the RSM model were used to estimate the several parameters analysed in this study (TPC and FC (including both colored anthocyanins and uncolored flavonols), as well as the radical scavenging activity (ABTS and DPPH) and reducing power (FRAP)) as a function of the independent variables (Table 2). To maximize each factor, the parameters were optimized (Table 3). Thus, the best combination for each parameter was reached at 60 °C, as the remaining variables were the pH obtained when supplementing the extractions solvent with 3.5% HCl to estimate all parameters. In what concerns the ethanol concentration, the optimum conditions based on the individual responses varied for each parameter: 69.6% for total phenolics, 55.1% for flavonoids, 90% for ortho-diphenols, 72.1% for ABTS, 53% for DPPH, and 65.8% for FRAP.

From these results, it was evidenced that the model was performed successfully and provided consistent results, not only because of the absolute error values, which ranged between 0.008–0.989 and are considered good values for sets of results ranging between 4.43 and 41.64 (Table 2), but also because of the proximity of the observed and predicted values (Table 1).

### 2.4. Quantitative Phenolic Profile via HPLC–DAD–ESI-MS/MS

In the HPLC–DAD–ESI-MS/MS analysis of the grape pomace extract, which was extracted under the best conditions retrieved from the application of the RSM methodology, fourteen compounds were identified, including phenolic acids, flavonols, and anthocyanins (Table 4).

The sample analysed consisted of not only skins but also seeds and some stems from two red grape varieties, ‘Sousão’ and ‘Touriga Nacional’. Thus, the predominance of the various compounds seems to depend on the constituents present in the sample, as well as on the grape varieties involved.

The HPLC–DAD–ESI–MS/MS screening of the grape pomace was performed, and fourteen compounds were identified and recorded between 321 and 520 nm.

The standard solutions were also infused in the mass spectrometer separately to obtain MS fragment ions. In the full scan mass spectra, the deprotonated molecular ions [M–H]^–^ of these compounds were stable and exhibited higher values (Table 1). Phenolic compound identification was based on the search of these ions, the interpretation of the collision-induced dissociation fragments, retention data, and comparison with data found in the literature [5].

Compound **1** showed a deprotonated molecular ion at an arbitrary mass unit (amu) of *m*/*z* 353, exhibiting a base peak at *m/z* 191, corresponding to deprotonated quinic acid, [quinic acid–H]^−^, and another characteristic ion at *m/z* 179 amu, [caffeic acid–H]^−^, in MS2 [38]. This allowed for its identification as 3-*O*-caffeoylquinic acid, which agrees with the phenolic alcohol profile described in this by-product [39].

The [M–H]^−^ ion at *m/z* 337 amu was detected, providing fragmentation base peaks at *m/z* 163 amu. This fragmentation yielded the MS2 base peak correspondent to the [p–coumaric acid–H]^−^ fragment, characteristic of 3-*p*-coumaroylquinic acid, which is in agreement with previous reports [38].

Regarding compound **3**, a degradation product of 5-*O*-caffeoylquinic acid gave the prominent [M–H]^−^ ion at *m/z* 353 amu in its ESI–MS spectrum. In general, for this compound, the [quinic acid–H]^−^ ion at *m/z* 191 amu appears as a MS2 spectrum base peak when the acyl group is linked to the 3-OH or 5-OH position; these two isomers can be further differentiated since the MS2 [caffeic acid–H]^−^ ion at *m/z* 179 amu is more significant for 3-OH compounds. In this sense, the present compound corresponds to 5-*O*-caffeoylquinic acid [40].

Apigenin pentoside ([M–H]^−^ at *m/z* 401 amu) (compound **4**) released an MS2 fragment at *m/z* 269 amu ([M–H–132]^−^, apigenin, indicating a loss of a pentosyl moiety).

Peak 5 demonstrated a deprotonated molecular ion at *m/z* 609 amu, resulting in a loss of 301 amu, corresponding to the loss of a hexose moiety, as previously reported [38].

Myricetin-*O*-rutinoside was found in the MS data along with its signal at *m/z* 625 amu and a fragment ion at *m/z* 317 amu, both corresponding to myricetin.

Regarding compound **7**, identified as another quercetin glycoside, it presented an [M–H]^−^ at *m/z* 301 amu, characteristic to the ion of quercetin aglycone.

Peak 8 displayed a molecular ion [M–H]^−^ at *m/z* 577 amu that provided two MS2 ion products at *m/z* 431 amu ([M–146]^−^, corresponding to the loss of a rhamnose moiety), and at *m/z* 285 amu ([M–146–146]^−^, obtained by the loss of two rhamnose moieties). Interestingly, the sequential loss of two rhamnoside moieties suggested a different location on the kaempferol molecule core for each sugar [41]. A compound with similar chromatographic and spectral characteristics was found by Shaheen et al. (2009) in *Tamus communis*, which was fully characterized by NMR [42].

Finally, peaks 9 and 10 were compounds identified as quercetin pentosides, since both presented a [M–H]^−^ at *m/z* 433 amu, yielding a base peak at *m/z* 301 amu, corresponding to the quercetin fragment [43].

Regarding the glycosylated anthocyanins identified in the grape pomace extract, it was observed that delphinidin-3-*O*-glucoside (peak 11), peonidin-3-*O*-glucoside (peak 12), and malvidin-3-*O*-glucoside (peak 13) presented [M–H]^−^ at *m/z* 465, 463, and 493 amu, respectively. The MS2 fragmentation pattern evidenced in all cases the loss of a glucose molecule [M–162]^+^ to yield protonated ions at *m/z* 303, 301, and 331 amu, respectively, which is in agreement with previous descriptions in the literature of the anthocyanin profile of grape by-products [43,44].

Finally, petunidin-3-(6″coumaroyl)-glucoside (peak 14) was also identified via the deprotonated molecular ion at *m/z* 625 amu, exhibiting a loss of coumaroylglucose moiety and acquisition of a water molecule [M–326 + 18]^+^, corresponding to MS2 fragments at *m/z* 317 amu.

In the analysis of the different constituents of pomace, skins, and pulp, and regarding anthocyanins, the predominant monoglucoside anthocyanin reported is malvidin in the skins and peonidin in the pulp [45]; in the extract analysed, peonidin was dominant and malvidin came immediately after with in terms of near values (0.0016 and 0.0013 mg/mL, respectively), respectively. Petunidin-3-*O*-(6-*O*-coumaroyl-glucoside) was also identified in the grape pomace extract under study [45]. In four grape pomace samples from different varieties, malvidin was again the predominant anthocyanin identified, but its concentration differs according to the variety. As in this work, petunidin-3-(6″coumaroyl)-glucoside was also detected in the four samples but in lower quantities compared to the remaining compounds (26–32 µg/g DW) [46]. Malvidin and delphinidin were identified as the predominant compounds among the various quantified compounds in the dyeing grape varieties. Peonidin is also among the anthocyanins identified in this variety, but is of a higher content in the pulp. Of the coumaroylated derivatives quantified, petunidin is present and its concentration belongs to the group with the highest values, after malvidin and peonidin [47]. Zhao et al. (2020) [44], Lingua et al. (2016) [48], and Trikas et al. (2016) [49] also identified the same four anthocyanins characterized in the present study in the red grape pomace of *V. vinifera* ‘Merlot’ grapes grown in China; of three red varieties (‘Syrah’, ‘Merlot’, and ‘Cabernet Sauvignon’) from Argentina; and those of a ‘Syrah’ variety, respectively. This last author determined similar concentrations of these compounds (from 4.47 to 955.85 mg/L) to those found in the present study (between 0.007 and 0.016 mg/mL).

Some authors have already found other phenolic compounds, namely non-anthocyanin flavonoids on grape pomace extracts. For instance, Maier et al. (2008) [50] and Peixoto et al. (2018) [51] have identified quercetin-3-*O*-glucoside in red grape pomace samples from Germany and Portugal, respectively, while Jara-Palacios et al. (2015) [52] identified quercetin-3-*O*-rutinoside in grape pomace of the variety Zalema, collected in Spain. Rockenbach et al. (2011) [39] and Ferri et al. (2017) [53] also identified another compound described in the present study, 3-*O*-caffeoylquinic acid (or chlorogenic acid), in the red grape pomace of a mixture of varieties cultivated in Brazil, as well as in white pomace derived from a mix of *V. vinifera* cv. ‘Trebbiano’ and ‘Verdicchio’ from Italy, with the latter showing in concentrations of 9.21 mg/L, which is significantly lower than the one determined in this study (1.082 ± 0.012 mg/mL).

## 3. Materials and Methods

### 3.1. Chemicals and Reagents

Ethanol absolute was purchased from Panreac (Castellar del Vallès, Barcelona, Spain) and hydrochloric acid, Follin-Ciocalteau, sodium carbonate, gallic acid, ABTS (2,2-Azino-bis(3-ethylbenzothiazoline-6-sulfonic)), DPPH (2,2-diphenyl-1-picrylhydrazyl), sodium acetate, Trolox (6-hydroxy-2,5,7,8-tetremethychroman-2-carboxylic acid), methanol, potassium persulfate, TPTZ (2,4,6-Tripyridyl-s-Triazine), and catechin from Sigma-Aldrich (St. Louis, MO, USA). The inorganic membrane filters of 0.22 µm were purchased from ANOTOP 10 plus and were from Whatman (Maidstone, UK). Aluminium chloride, sodium nitrite, sodium hydroxide, and iron (III) chloride were from Merck (Darmstadt, Germany). Acetic acid was purchased from Panreac (Panreac Química S.L.U., Barcelona, Spain). The microplates used were commercialized by Frilabo (Milheirós, Portugal), and the water used in all assays was treated with the SGS^TM^ water purification system.

### 3.2. Plant Material

Grape (*Vitis vinifera*) pomace from the red varieties ‘Touriga Nacional’ and ‘Sousão’ corresponded to the 2020 season. The by-product was provided by a local producer from the North of Portugal, in the region of Vinhos Verdes (Maia; 41°14′45.1″ N and 8°33′7.2″ W). After collection, the material was stored at −20 °C until freeze-drying. The dried material was ground to a fine powder and stored protected from light for phenolic extraction and analysis.

### 3.3. Extraction Procedure

The dried samples (40 mg) were processed according to diverse extracting conditions designed according to the different levels of the variables referred in Table 5 with 1.5 mL of solvent (ratio ~1:40, *w/v*).

The ethanol used contained different proportions of hydrochloric acid to obtain a distinct pH that was hypothesized to influence the efficiency of the polyphenol extraction according to Periago et al. (2002) [54]. Extractions were performed in a water bath for 30 min using different temperatures. Then, the extracts were centrifuged at 5000 rpm for 15 min and at 4 °C (Sigma 2-16K, Osterode am Harz, Germany). The supernatants were collected into a volumetric flask of 5 mL. The solid residue was extracted two more times under matching conditions, and the extracts were pooled in the volumetric flask. The repeated extractions were performed to overwhelm possible saturations of the extracting solvent and thus, guarantee the attainment of all the polyphenolic burden of the grape pomace. The final volume was made up to 5 mL to reduce the variability between samples attributable to dilution issues. Finally, the extract was filtered through a 0.22-µm inorganic membrane filter (ANOTOP 10 plus, Whatman, Maidstone, UK) and preserved at 4 °C until analysis.

### 3.4. Experimental Design

A Box–Behnken design was applied to optimize the best extraction conditions for the polyphenolic fraction of grape pomace. The experiment carried out at different levels of food quality ethanol concentration, pH, and temperature (Table 5) was designed to retrieve the best extraction conditions for grape pomace polyphenols concerning total phenolic content (TPC) and flavonoid content (FC), along with the ABTS and DPPH radical scavenging capacity, and FRAP-based reducing power (*n* = 27, Table 1).

Preliminary assessments were performed to obtain an adequate range of values for the considered factors (*X*1 (percentage of ethanol): 50%, 70%, and 90%; *X*2 (pH level based on the percentage of HCl): 0.5%, 2.0%, and 3.5% HCl; and *X*3 (Temperature): 20, 40, and 60 °C). The pretests set up to obtain the adequate range for the considered factors were designed based on previous reports assessing the effect of the extraction conditions on the polyphenolic burden of grape pomace extracts [17,18], which provided the estimated reference to establish the central point from which the symmetric distribution of the alcohol percentage, HCl percentage, and temperature were built. The results retrieved evidenced that data obtained upon the Response Surface Methodology (RSM) approach was successfully applied for optimal estimation of the different variables. The central point for the extraction of phytochemicals compounds follows the symmetric distribution of the solvent percentage, pH, and temperature (*X*1: 70%, *X*2: 2.0, *X*3: 40 °C, respectively).

### 3.5. Total Phenolics and Flavonoids

The total phenolic (Folin–Ciocalteau method) and flavonoid (aluminum chloride complexation assay) content of the grape pomace samples were determined via spectrophotometric analyses adapted to a microplate scale according to the methodology described in the literature [29,55], using a spectrophotometric Multiscan microplate reader (Thermo Scientific Multiskan GO Microplate Spectrophotometer, Vantaa, Finland). Regarding the total phenols determination, 20 µL of standard solutions and respective samples were added directly to each well, followed by 100 µL of the Folin–Ciocalteau sample and then 80 µL of sodium carbonate (Na_2_CO_3_) 7.5% were added. The changes in the absorbance were monitored at 750 nm after 60 min of reaction at room temperature.

For the assessment of samples in terms of the flavonoid’s content, in each well 24 µL of standard solutions and respective samples were added, plus 28 µL of sodium nitrate (NaNO_2_) (50 g/L), and then incubated for 5 min at room temperature. Afterwards, 28 µL of aluminium chloride (100 g/L) was added, and 6 min later, 120 µL of sodium hydroxide 1 M was added, and the absorbance was monitored immediately at 510 nm.

The concentrations of the total phenolics and flavonoids were set up by resorting to gallic acid and catechin calibration curves (5–200 mg/L), correspondingly, freshly prepared each day of analysis and expressed in milligrams of gallic acid equivalents per gram of dry weight (mg GAE/g dw) and milligrams of catechin equivalents per gram of dry weight (mg CE/g dw).

### 3.6. Radical Scavenging Capacity

The antioxidant capacity of the grape pomace extracts was determined via DPPH and ABTS radical scavenging tests, as well as the FRAP-based determination of the reducing capacity. All the methodologies were adapted to a microscale [56]. Spectrophotometric reads were developed at 520, 734, and 593 nm, respectively. In all the methodologies, a Multiscan FC microplate reader (Thermo Scientific Multiskan GO Microplate Spectrophotometer, Vantaa, Finland) was used, and the results were expressed as millimoles of Trolox equivalent antioxidant capacity per gram of dry weight (mmoles TEAC/g dw).

### 3.7. HPLC-DAD-ESI-MS/MS Analysis

The phenolic profile of the pomace samples was performed using a High-Performance Liquid Chromatography-Diode Array Detector (HPLC-DAD), using Gilson HPLC (Villers-le-bel, France) and Finnign/Surveyor DAD (Thermo Electron, San Jose, CA, USA) immediately after the solid–liquid extraction of the sample, according to the method described by Aires and Carvalho (2020) [57]. In the present work, chromatographic separation was performed using a C18 column (250 × 4.6 mm, 5 µm, ACE HPLC Columns, Advanced Chromatography Technologies Ltd., Abeerden, Scotland, UK), with water/trifluoroacetic acid (99.9:0.1, *v/v*) as solvent A and acetonitrile/trifluoroacetic acid (99.9:0.1, *v/v*) as solvent B. The linear gradient used started from 0% solvent B at 0 min, 0% solvent B at 5 min, 20% solvent B at 15 min, 50% solvent B at 30 min, 100% solvent B at 45 min, 100% solvent B at 50 min, 0% solvent B at 55 min, and 0% solvent B at 60 min, with a flow rate of 1 mL/min, at room temperature, and an injection volume of 20 µL. All samples were analyzed in triplicate (*n* = 3). Chromatograms were recorded between 320 and 520 nm for the specific determination of the different classes of phenolic compounds. Considering various parameters such as peak retention time, UV max absorbance bands, and UV spectra, identification of the compounds was accomplished via comparison with external authentic standards (Extrasynthese, Genay Cedex, France). The analysis of the reference standards, previously prepared in a methanol/water (70:30, *v/v*) solution at a concentration of 1.0 mg/mL, was performed in HPLC-DAD. The internal standard method was employed to quantify the amount of each identified polyphenol. Concentrations were expressed in mg/g of dry weight (mg/g dw). The HPLC system was coupled to an ion trap mass spectrometer (ultra HCT Bruker, Bremen, Germany) equipped with electrospray ionization (ESI), and operated in a negative ion mode. Data acquisition and processing were accomplished using the B.01.03-SR2 software for ChemStation for an LC-3D system from Agilent Technologies (Waldbronn, Germany), as previously described [56]. The capillary and voltage were maintained at 350 °C and 4 kV, respectively. Mass scan and daughter spectra were measured from *m*/*z* 100 to 1500. Collision-induced fragmentation experiments were executed in an ion trap, using helium as collision gas and setting the collision energy at 50% [58].

### 3.8. Statistical Analysis

According to the variables under study, for each assay, the means and standard deviations were calculated separately using Statgraphics Centurion XVI (StatPoint Technologies Inc., Warrenton, VA, USA), as well as the coefficients for the equations generated by the model. The one-way analysis of variance (ANOVA) was also performed. The regression coefficients were determined and when statistically significant differences were observed, a multiple Tukey range test was run out. Statistical significance was set up at *p* < 0.05. All statistical analyses were performed using SPSS 21.0 software (LEAD Technologies, Inc., Chicago, IL, USA).

## 4. Conclusions

The optimization procedure developed to maximize the extraction of grape pomace’s polyphenols via the RSM used quadratic models to predict the response during the extraction procedure to the independent variables considered (the percentage of ethanol, the pH, and the extraction temperature). As a result of this experimental approach, it achieved an effective model for estimating the effect of the three independent variables on the extraction of phenolic compounds present in grape pomace, as well as for achieving the optimal radical scavenging capacity and reducing power. The analysis of the different variables under study evidenced an interesting trend concerning pH and temperature used, which were identified as the most appropriate extraction conditions for achieving the objective-outlined 60 °C, a percentage of HCl of 3.5%, and percentages of ethanol ranging between 53.0 and 72.1%. Not forgetting sustainability, the good results obtained when using higher temperatures also allow for a reduction in extraction time. The different conditions retrieved are compatible with the scale up of this extraction methodology to sectorial industries, for instance, in the field of bioactive ingredients of technological or biological uses. Alternatively, for the direct uses and the contribution of these results to enhancing the diversity of socioeconomic activities based on valorizing up-to-date, under exploited, and pollutant materials, and keeping in mind the goal of a circular economy, the choice of ethanol as a solvent also allows, in turn, its application in the food industry, given it is a non-toxic and environmentally friendly solvent. The overall consideration of this results jointly with previous optimizations focused on fine-tuning the setting for solvent concentration, extraction time, type of solvent, and the influence of the liquid/solvent ratio allows us to obtain a complete picture of the diverse factors affecting the efficiency of polyphenols extraction, and thus dedicate further efforts in the applicability of this matrix and its polyphenolic burden to the characterization of the biological scope in the frame of different pathophysiological conditions, with a particular emphasis on allowing us to advance toward formulations that lead to practical application and the utilization of their well-characterized functionality.

## Figures and Tables

**Table 1:** Effect of processing variables on the (poly)phenolic content, radical scavenging capacity, and reducing power of hydroethanolic extracts of grape pomace using the Response Surface Methodology.

Assay	Coded Level			TPC (mg GAE/g dw)		FC (mg CE/g dw)		ABTS (mmol TEAC/g dw)		DPPH (mmol TEAC/g dw)		FRAP (mmol TEAC/g dw)	
	Ethanol Concentration (%)	pH (% of HCl)	Temperature (°C)	Observed	Predicted	Observed	Predicted	Observed	Predicted	Observed	Predicted	Observed	Predicted
1	−1 (50)	−1 (0.5)	−1 (20)	8.84	8.49	5.93	5.18	0.09	0.10	0.09	0.10	0.11	0.11
2	−1 (50)	−1 (0.5)	0 (40)	15.77	14.81	8.49	7.45	0.15	0.14	0.15	0.14	0.15	0.14
3	−1 (50)	−1 (0.5)	1 (60)	31.88	31.72	15.11	16.77	0.25	0.25	0.25	0.25	0.25	0.25
4	−1 (50)	0 (2.0)	−1 (20)	10.56	11.64	6.38	5.84	0.12	0.12	0.12	0.12	0.12	0.12
5	−1 (50)	0 (2.0)	0 (40)	18.34	19.85	8.83	8.75	0.15	0.16	0.15	0.16	0.17	0.17
6	−1 (50)	0 (2.0)	1 (60)	38.06	38.64	18.46	18.70	0.27	0.27	0.27	0.27	0.29	0.30
7	−1 (50)	1 (3.5)	−1 (20)	11.97	10.21	6.35	6.87	0.12	0.11	0.12	0.11	0.12	0.12
8	−1 (50)	1 (3.5)	0 (40)	19.59	20.31	10.41	10.41	0.17	0.16	0.17	0.16	0.20	0.18
9	−1 (50)	1 (3.5)	1 (60)	41.64	40.98	7.40	21.00	0.28	0.28	0.28	0.28	0.31	0.32
10	0 (70)	−1 (0.5)	−1 (20)	11.87	12.04	5.43	6.61	0.11	0.10	0.11	0.10	0.11	0.11
11	0 (70)	−1 (0.5)	0 (40)	16.75	17.53	7.47	8.44	0.13	0.13	0.13	0.13	0.14	0.14
12	0 (70)	−1 (0.5)	1 (60)	34.71	33.62	18.37	17.33	0.24	0.24	0.24	0.24	0.25	0.25
13	0 (70)	0 (2.0)	−1 (20)	14.63	15.79	5.24	6.91	0.11	0.12	0.11	0.12	0.12	0.13
14	0 (70)	0 (2.0)	0 (40)	23.91	23.17	10.75	9.39	0.15	0.16	0.15	0.16	0.19	0.18
15	0 (70)	0 (2.0)	1 (60)	41.72	41.14	20.11	18.90	0.28	0.27	0.28	0.27	0.31	0.30
16	0 (70)	1 (3.5)	−1 (20)	16.38	14.96	7.34	4.89	0.13	0.13	0.13	0.13	0.12	0.13
17	0 (70)	1 (3.5)	0 (40)	22.48	24.21	11.15	1070	0.17	0.18	0.17	0.18	0.19	0.18
18	1 (70)	1 (3.5)	1 (60)	44.93	40.71	22.95	20.09	0.30	0.29	0.30	0.29	0.36	0.31
19	1 (90)	−1 (0.5)	−1 (20)	8.74	9.15	7.38	7.42	0.06	0.07	0.06	0.07	0.08	0.08
20	1 (90)	−1 (0.5)	0 (40)	15.03	13.82	9.27	8.83	0.11	0.10	0.11	0.10	0.12	0.11
21	1 (90)	−1 (0.5)	1 (60)	26.67	29.08	17.85	17.28	0.19	0.21	0.19	0.21	0.21	0.22
22	1 (90)	0 (2.0)	−1 (20)	12.77	13.49	8.16	7.38	0.11	0.10	0.11	0.10	0.12	0.10
23	1 (90)	0 (2.0)	0 (40)	24.9	20.15	9.53	9.41	0.16	0.15	0.16	0.15	0.15	0.15
24	1 (90)	0 (2.0)	1 (60)	36.07	37.19	16.31	18.50	0.26	0.26	0.26	0.26	0.27	0.28
25	1 (90)	1 (3.5)	−1 (20)	13.26	13.25	9.28	7.70	0.12	0.12	0.12	0.12	0.11	0.10
26	1 (90)	1 (3.5)	0 (40)	18.66	21.69	7.84	10.38	0.16	0.17	0.16	0.17	0.14	0.17
27	1 (90)	1 (3.5)	1 (60)	38.11	40.71	18.48	20.08	0.28	0.29	0.28	0.29	0.28	0.31

CE catechin equivalent; GAE, gallic acid equivalent; FC, flavonoids content; TE, Trolox equivalent; TPC, total phenolic compounds.

**Table 2:** Corresponding *F*-values and *p*-values for each obtained coefficient and second-order polynomial models were used to express the content in total phenolics (TPC) and flavonoids (FC), as well as the ABTS, DPPH, and FRAP-based antioxidant and reducing activities as a function of independent variables in grape pomace.

Variable	Statistic	*X*1	*X*2	*X*3	*X*1*,*2	*X*1*,*3	*X*2*,*3	*X*1^2^	*X*2^2^	*X*3^2^	Model *F*-Value
TPC	*p*-value	0.851	***	***	0.358	0.205	*	**	*	***	0.16
	*F*-value	0.04	39.58	569.91	0.89	1.74	8.32	11.26	6.31	33.61	
FC	*p*-value	0.41	*	*	0.49	0.40	0.25	0.66	0.79	***	0.20
	*F*-value	0.71	7.41	209.99	0.51	0.76	1.45	0.20	0.08	27.46	
ABTS	*p*-value	**	***	***	*	0.652	0.063	0.070	0.078	***	0.59
	*F*-value	8.86	73.93	858.08	12.73	0.21	3.94	3.74	3.52	59.27	
DPPH	*p*-value	***	***	***	0.539	*	0.088	0.121	0.287	**	0.89
	*F*-value	32.34	208.97	270.04	0.40	6.79	3.36	2.72	1.23	15.82	
FRAP	*p*-value	*	***	***	0.428	0.998	**	0.072	0.141	***	0.05
	*F*-value	6.17	30.14	384.69	0.66	0.01	10.75	3.68	2.38	23.94	
Polynomial model										R^2^	MAE
TPC = 23.1678 + 0.0988027X1 + 3.33974X2 + 12.6736X3 − 3.21974X1^2^ + 0.591537X1X2 − 0.825963X1X3 − 2.29359X2^2^ + 1.88461X2X3 + 5.29474X3^2^										0.962	0.010
FC = 9.38469 + 0.333288X1 + 1.12598X2 + 5.99543X3 − 0.305237X1^2^ − 0.355901X1X2 − 0.435901X1X3 + 0.184871X2^2^ + 0.636473X2X3 + 3.5232X3^2^										0.909	0.989
ABTS = 0.161795 − 0.00761641X1 + 0.0226204X2 + 0.0770648X3 − 0.0092011X1^2^ + 0.0110754X1X2 − 0.00142462X1X3 − 0.00849074X2^2^ + 0.00643056X2X3 + 0.0348426X3^2^										0.975	0.008
DPPH = 0.2413529 − 0.0218333X1 + 0.0690476X2 + 0.0784921X3 + 0.010625X1^2^ + 0.00308333X1X2 − 0.01275X1X3 − 0.00761905X2^2^ + 0.0119048X2X3 + 0.027381X3^2^										0.958	0.100
FRAP = 0.178926 − 0.0105464X1 + 0.0239717X2 + 0.0856384X3 − 0.0151381X1^2^ + 0.0041814X1X2 + 0.0000137318X1X3 − 0.0115839X2^2^ + 0.0176242X2X3 + 0.0367495X3^2^										0.945	0.010

Significant at *p* < 0.05 (*), *p* < 0.01 (**), and *p* < 0.001 (***). *X*1: Ethanol concentration (%), *X*2: pH (%HCl), and *X*3: Temperature (°C). MAE: Mean absolute error; R^2^, regression coefficient.

**Table 3:** Predicted values under optimum conditions based on the individual responses.

Response	Process Variables			Predicted Values at the Optimal Conditions
	Ethanol Concentration (%)	HCl Concentration (%)	Temperature (°C)	
TPC (mg GAE/g dw)	69.6	3.5	60.0	44.066
FC (mg CE/g dw)	55.1	3.5	60.0	21.022
ABTS (mmol TEAC/g dw)	72.1	3.5	60.0	0.294
DPPH (mmol TEAC/g dw)	53.0	3.5	60.0	0.456
FRAP (mmol TEAC/g dw)	65.8	3.5	60.0	0.332

**Table 4:** Identification of phenolic compounds in grape pomace extract via HPLC–DAD–MSn in negative mode.

Peak	Rt	λmax	[M–H]^−^ *m/z*	MS2 [M–H]^−^ (Relative Abundance)	Tentative Identification	Concentration (mg mL^−1^)
1	4.08	321	353	191 (100), 179 (61), 173 (4), 161 (8), 135 (17)	3-*O*-Caffeoylquinic acid	1.082 ± 0.012
2	5.12	322	337	163 (100)	*p*-Coumaroylquinic acid	0.072 ± 0.002
3	5.26	321	353	191 (100), 179 (23), 173 (31), 161 (9)	5-*O*-Caffeoylquinic acid	0.059 ± 0.001
4	5.75	335	401	269 (100)	Apigenin-*O*-pentoside	0.104 ± 0.004
5	13.18	343	609	301 (100)	Quercetin-3-*O*-rutinoside	0.377 ± 0.002
6	13.76	348	625	317 (100)	Myricetin-*O*-rutinoside	0.203 ± 0.002
7	14.39	342	463	301 (100)	Quercetin-3-*O*-glucoside	0.097 ± 0.002
8	14.94	336	577	431 (36), 285 (100)	Kaempferol 3′,4′-di-*O*-rhamnoside	0.043 ± 0.002
9	16.24	347	433	301 (100)	Quercetin-*O*-pentoside	0.312 ± 0.003
10	16.68	349	433	301 (100)	Quercetin-*O*-pentoside	0.059 ± 0.002
11	18.35	520	465	303 (100)	Delphinidin-3-*O*-glucoside	0.007 ± 0.000
12	19.57	520	463	301 (100)	Peonidin-3-*O*-glucoside	0.016 ± 0.001
13	20.71	520	493	331 (100)	Malvidin-3-*O*-glucoside	0.013 ± 0.001
14	25.35	520	625	317 (100)	Petunidin-3-(6″coumaroyl)-glucoside	0.008 ± 0.000
					Total Phenolic acid	1.212 ± 0.015
					Total Flavonoids	1.195 ± 0.017
					Total Anthocyanins	0.044 ± 0.002
					Total Phenolic compounds	2.407 ± 0.032

Rt—retention time.

**Table 5:** Symbols and coded factor levels for the considered independent variables.

Independent Variables	Code	Levels		
		−1	0	1
Ethanol concentration (%)	*X*1	50	70	90
pH (% of HCl)	*X*2	0.5	2.0	3.5
Temperature (°C)	*X*3	20	40	60

## Data Availability

Not applicable.
